# Moving Away from Amyloid Beta to Move on in Alzheimer Research

**DOI:** 10.3389/fnagi.2015.00002

**Published:** 2015-01-22

**Authors:** María G. Moreno-Treviño, Jesús Castillo-López, Irene Meester

**Affiliations:** ^1^Department of Basic Sciences, Universidad de Monterrey, San Pedro Garza García, Mexico; ^2^Department of Psychology, Health Division, Universidad de Monterrey, San Pedro Garza García, Mexico

**Keywords:** Alzheimer’s disease, amyloid beta, bioenergetics, etiology, herpes simplex type 1

Alzheimer’s disease (AD) is characterized by a progressive decay of cognitive abilities, most remarkably (spatial) memory and learning. AD is diagnosed by clinical mental tests, often combined with the detection of neurobiological markers, mainly brain imaging studies and a decreased amyloid beta (Aβ) level and/or increased total and hyper-phosphorylated Tau protein (tau-P) in cerebral spinal fluid (Hampel et al., 2008; Alzheimer’s Association, 2014). The diagnosis is confirmed post-mortem by histopathological detection of senile plaques, composed of Aβ accumulations, and tau-P-containing neurofibrillary tangles (Jellinger and Bancher, 1998). However, non-demented, aged patients may have a histopathology that is indistinguishable from AD (Price and Morris, 1999; Nelson et al., 2012). Furthermore, the brains of AD may have additional changes, such as (micro)vascular changes (Scheibel et al., 1989; de la Torre, 2002; Bell and Zlokovic, 2009; Hommet et al., 2011), white matter hyperintensities (Kandiah et al., 2015), and vacuolar cells, which are not considered as pathognomonic features under current standards (Nelson et al., 2012).

## Weaknesses of the Amyloid Beta Cascade Hypothesis

In the last two decades, AD-related investigation has absorbed approximately 18% of neuroscience research efforts (Sorensen, 2009), but so far neither a cure nor an effective method of prevention has been formulated. If Alzheimer etiology is multifactorial and complex, how can one justify that etiology studies are dominated by a single hypothesis, the Aβ cascade (ABC) hypothesis (Hardy and Higgins, 1992; Selkoe, 2008)? Central in an actualized version of this hypothesis is the formation of soluble oligomer Aβ forms (Selkoe, 2008), which cause synaptic dysfunction, tau-P-containing neurofibrillary tangles (Busciglio et al., 1995), and progressive cognitive decline. The ABC hypothesis was reinforced by the identification of gene defects in *APP*, *PSEN1*, and *PSEN2* in patients with an early-onset, inherited form of the disease, and further strengthened when transgenic mouse models that express familial human *APP* and *PSEN* mutations recapitulate most features of human disease. The *APP* gene encodes the Aβ precursor protein (APP), from which Aβ is liberated after step-wise, amyloidogenic, proteolytic processing. The *APP* gene is located on chromosome 21, which may explain early-onset AD in Down’s syndrome patients. The genes for *PSEN1* and *PSEN2* encode presenilin 1 and 2, which are part of the γ-secretase complex, the enzyme that carries out the second cleavage in APP processing (Haass et al., 2012). Surprisingly, genome-wide association studies have not identified mutations for either enzyme that performs initial APP cleavage: (1) α-secretase, in the non-amyloidogenic route, and (2) β-secretase/BACE1, in the amyloidogenic route. Notwithstanding, the ABC hypothesis does not consider another APP metabolite to be the toxic agent (Roher et al., 1991), though the APP intracellular domain seems to have neurodegenerative potential (Chang et al., 2006; Ghosal et al., 2009). Furthermore, the functions of APP and APP-derived fragments, including Aβ that is also produced during homeostasis (Haass et al., 2012), have not yet been elucidated. Meanwhile, numerous *in vitro* and animal studies have strengthened the ABC hypothesis by linking Aβ with tau-P, formation of neurofibrillary tangles, neurotoxicity, and cognitive defects (Selkoe, 2008). It should be noted, though, that a large quantity of supporting data were obtained from experiments with serious flaws in experimental design, for example: (1) the application of non-physiological high concentrations of Aβ (Selkoe, 2008; McGeer and McGeer, 2013), (2) the lack of proper negative controls, which tend to be limited to the solvent or vehicle, but do not include alternative fragments of APP or a “mock” protein) (Busciglio et al., 1995; Pentreath and Mead, 2004; Boyd-Kimball et al., 2005), (3) the absence of Aβ elimination mechanisms in cell culture studies, and (4) the absence of mentally healthy human controls in some studies (Cummings et al., 2014).

In spite of the aforementioned weaknesses, the ABC hypothesis may explain heritable, early-onset AD. However, the assumption that the hypothesis can be extrapolated to sporadic, late-onset AD may be a mistake. Arguments that similar clinical symptoms and neurobiological findings justify such extrapolation are not supported by other diseases with a differential onset time. For example, skin blistering diseases may have similar clinical and histological features, but the early-onset forms (epidermolysis) have a genetic basis, whereas the late-onset forms (pemphigoids) are autoimmune diseases (Valeski et al., 1992). Only for the latter, a treatment based on etiology is available. Likewise, it is not surprising that Alzheimer treatments that target components of the ABC hypothesis, though promising in animal models of early-onset Alzheimer, fail in human clinical studies (Geerts et al., 2013), where 95% of the patients suffer late-onset AD (Alzheimer’s Association, 2014; Hill et al., 2014). Furthermore, the ABC hypothesis ignores certain neurobiological aspects. First, senile plaques and neurofibrillary tangles display dissimilar spatiotemporal distribution (Jucker and Walker, 2011). If the former induced the latter, similar patterns would be expected. Second, the role of glial cells in the ABC hypothesis is reduced to an Aβ elimination route and does not consider their recently recognized participation in all essential neurological tissue functions (Kettenmann et al., 2011; Oberheim et al., 2012). Cell counting studies on human Alzheimer brains have focused on neurons (Pelvig et al., 2003), but the possible loss of glial cell extensions or cells, or a change in their functioning, has hardly been appreciated. Third, if Aβ were as toxic as claimed, how can one explain cognitive health in subjects that contain Aβ accumulations that would credit Alzheimer disease? Indeed, it is amply recognized that an Aβ plateau is reached long before clinical symptoms may appear (Braak and Del Tredici, 2011; Cummings et al., 2014). Proponents of the ABC hypothesis provide several explanations: (1) the phenomenon reflects the prodromal period, (2) cognitively healthy subjects have a “cognitive reserve” (Stern, 2012), i.e., excess of dendritic spines, or (3) the inert and very resistant senile plaques should be considered rather protective than toxic. Surprisingly, the possibility that another APP metabolite may be the toxic component is not considered in the ABC hypothesis. Besides, the accumulation of tau-P, whatever may have induced its production, associates better with clinical symptoms and decline than Aβ (Cummings et al., 2014). Still, neurofibrillary tangles are not specific either to AD as they occur in a variety of neurodegenerative diseases (Nelson et al., 2012). Fourth, the ABC hypothesis does not pay attention to the fact that the hippocampus is the site of both disease initiation and adult neurogenesis (Wang et al., 2014). Finally, the ABC hypothesis ignores typical characteristics of the disease, such as the spatiotemporal pattern of affected brain areas, the presence of white matter hyperintensities (Kandiah et al., 2015), and vacuolated cells (Nelson et al., 2012). Alternative hypotheses, in which Aβ may occur as a symptom, but not as the main cause, are presented below.

## Promising Alternative Etiology Hypotheses

In the bioenergetics hypothesis, low (sex) steroid hormone levels affect glucose transport into the brain, as has been demonstrated in animal models (Yao and Brinton, 2012). In order to fulfill its high energy demands, the brain switches first to a ketone body-based metabolism and later to fatty acid oxidation. This process is accompanied by oxidative stress, a decline in mitochondrial function, Ca^2+^ overload, and general cellular malfunction (Camandola and Mattson, 2011). These changes in metabolism would affect the white matter either by inadequate myelin synthesis or by increased myelin degradation by astroglia, which could turn them into vacuolated cells. Indeed, wells with lipoid granules were noted as a pathological hallmark by Alois Alzheimer, but they have been ignored by many scientists in the field (Di Paolo and Kim, 2011). White matter degeneration increases free cholesterol, which may be incorporated in membranes and lead to an increased number of lipid rafts. Lipid rafts favor the amyloidogenic processing of APP. In this scenario, Aβ is rather a down-stream symptom than a cause of disease. Estrogen-based therapy, initiated around the menopause transition in subjects with healthy, non-compromised brains, has been associated with a decreased risk of AD (Yao and Brinton, 2012). Furthermore, there are other neurosteroid hormones that are neuroprotective by improving bioenergetics, increasing anti-oxidant activity (Grimm et al., 2014), or promoting neurogenesis, and as such may provide new therapeutic options for AD patients. Indeed, some are currently studied in clinical trials.

Another hypothesis, the reactivation of latent *Herpes simplex 1* (RL-HSV), was suggested to explain the earliest predilection of entorhinal and hippocampal nervous tissue to display AD-related cognitive decline, the same brain regions that are primarily affected because of herpes simplex encephalitis (Ball et al., 2013). However, most people infected with *H. simplex* 1 (HSV-1) only develop a temporary cold sore, followed by a life-lasting, latent infection in the trigeminal ganglia. Periodic reactivation may be asymptomatic, unless the immune system is weakened, as in old age. The bipolar trigeminal ganglion neurons branch centrifugally to distal nerve endings, and centripetally to mesencephalic nuclei and locus coeruleus, where earliest tau pathology has been observed (Braak and Del Tredici, 2011). From there, dissemination could pass further into the cerebral cortex (Armien et al., 2010). Vacuolated cells could represent infected neurons or glial cells in process of degeneration (Ohara et al., 2000). HSV-1 binding to neuronal membranes causes persistent hyper-excitability and increased intracellular Ca^2+^ (Piacentini et al., 2014), which is thought to contribute to neurodegeneration in the calcium hypothesis (Mattson, 2010). Viral encoded kinases, such as UL13, could phosphorylate human tau protein by cross-species substrate specificity (Geiss et al., 2004). In this model also, tau and Aβ pathology would be rather a consequence than a cause of AD. HSV-1 DNA has been located within senile plaques (Itzhaki, 2014). Anti-viral drug reduced Aβ, tau-P, and HSV-1 accumulation in cell cultures infected with HSV-1 (Itzhaki, 2014). HSV-1 prevalence, tested by seropositivity, is high (50–80%) in most countries, and tends to increase with age (Smith and Robinson, 2002). Indirect evidence for the reactivation of HSV-1 comes from a prospective study in which anti-HSV-1 IgM-seropositivity, a marker of primary infection or reactivation of latent infection, highly correlated with the development of late-onset AD (Letenneur et al., 2008). *APOE4*, a genetic risk factor for late-onset AD, facilitates viral infectivity (Burgos et al., 2006). Many interesting viral/host interactions that target to AD susceptibility genes have been discovered (Carter, 2008), which provide an integrated, convincing support to the RL-HSV hypothesis for late-onset AD etiology. Additionally, AD brains contain clear evidence for activation of the inflammatory pathway. Chronic inflammation has been proposed as the main initiator of late-onset AD in the inflammatory hypothesis (Morris et al., 2014), but it might reflect a reaction to an infection (Hill et al., 2014). A reactivation of HSV-1 would have to be different from known lytic cycle replication as AD patients do not suffer from encephalitis or cold sores. Likewise, no direct evidence of HSV-1 reactivation, such as the detection of increased levels of lytic transcripts or proteins, has been convincingly reported. Recently, the axonal transport of nude HSV-1, i.e., a viral particle with capsid but without envelope or associated glycoproteins, has been reported (Wisner et al., 2011). This phenomenon may explain negative or low HSV-1 immunoreactivity on Alzheimer’s brain if antibodies or antiserum to envelope proteins were used. Alternatively, other (neurotropical) pathogens may be responsible (Lurain et al., 2013). Besides, the reactivation of a neurotropic pathogen may have detonated an autoimmune disease, originally initiated by cross-reactivity between pathogen and host antigens. Interestingly, intensive immunoreactivity to human antibody has been reported in Alzheimer’s brains (D’Andrea, 2003), which was interpreted as a sign of autoimmune disease. The cognate antigen has not been identified, and could be either an auto-antigen or a pathogen-derived antigen. Medical history provides examples of diseases that used to be attributed to age, stress, or chronic inflammation until pathogen involvement could be proven. Nowadays, we know that peptic ulcers are caused by *Helicobacter pylori* (Marshall and Warren, 1984) and that periodontal disease and concomitant tooth loss are caused by a limited number of oral pathogens (Cugini et al., 2013). As a result, prevention methods and antibiotic treatments have come available.

In our opinion, now that the ABC hypothesis road seems dead-ended, alternative roads deserve to be explored with more effort in order to discover new targets for diagnostics, cure, and prevention.

## Author Contributions

All authors participated in the conception to publish this opinion in order to promote funding for alternative Alzheimer research. María G. Moreno-Treviño, Jesús Castillo-López, and Irene Meester contributed equally to information acquisition. Irene Meester drafted the work, which was critically reviewed by María G. Moreno-Treviño and Jesús Castillo-López. All authors have approved the submitted version and declare accountability for the accuracy and integrity of the document.

## Conflict of Interest Statement

The authors declare that the research was conducted in the absence of any commercial or financial relationships that could be construed as a potential conflict of interest.
